# Ultrasound combined with microbubble mediated immunotherapy for tumor microenvironment

**DOI:** 10.3389/fphar.2024.1304502

**Published:** 2024-02-29

**Authors:** Yunfeng Wu, Jiajia Li, Linfeng Shu, Zhaoyu Tian, Siru Wu, Zuohui Wu

**Affiliations:** Department of Ultrasound, Affiliated Hospital of Zunyi Medical University, Zunyi, China

**Keywords:** ultrasound, cavitation effect, tumour microenvironment, immunotherapy, tumor cell

## Abstract

The tumor microenvironment (TME) plays an important role in dynamically regulating the progress of cancer and influencing the therapeutic results. Targeting the tumor microenvironment is a promising cancer treatment method in recent years. The importance of tumor immune microenvironment regulation by ultrasound combined with microbubbles is now widely recognized. Ultrasound and microbubbles work together to induce antigen release of tumor cell through mechanical or thermal effects, promoting antigen presentation and T cells’ recognition and killing of tumor cells, and improve tumor immunosuppression microenvironment, which will be a breakthrough in improving traditional treatment problems such as immune checkpoint blocking (ICB) and himeric antigen receptor (CAR)-T cell therapy. In order to improve the therapeutic effect and immune regulation of TME targeted tumor therapy, it is necessary to develop and optimize the application system of microbubble ultrasound for organs or diseases. Therefore, the combination of ultrasound and microbubbles in the field of TME will continue to focus on developing more effective strategies to regulate the immunosuppression mechanisms, so as to activate anti-tumor immunity and/or improve the efficacy of immune-targeted drugs, At present, the potential value of ultrasound combined with microbubbles in TME targeted therapy tumor microenvironment targeted therapy has great potential, which has been confirmed in the experimental research and application of breast cancer, colon cancer, pancreatic cancer and prostate cancer, which provides a new alternative idea for clinical tumor treatment. This article reviews the research progress of ultrasound combined with microbubbles in the treatment of tumors and their application in the tumor microenvironment.

## 1 Introduction

The tumour microenvironment (TME) is a complex and rich multicellular environment for tumor growth, which is composed of tumor cells, immune cells, stromal cells, extracellular matrix (ECM), etc (Bilotta et al., 2022). In recent years, the importance of TME in dynamically regulating the progress of cancer and influencing treatment results has been widely recognized, and the rapid development of tumor immunotherapy against TME has established it as an important treatment method for various cancers. Compared with surgery, radiotherapy and chemotherapy, tumor immunotherapy can stimulate the body’s immune system and enhance the body’s immune defense mechanism against tumors, thus indirectly attacking tumor cells and reshaping the immune microenvironment (Xu et al., 2019; Kumar et al., 2021). On the one hand, immune-mediated tumor cell death can be enhanced by promoting immune tumor cells to recognize and eliminate target cells carrying tumor antigens, and on the other hand, immune suppression signals induced by tumor cells can be eliminated or reduced (Zimmermannova et al., 2021).

At present, a variety of immunotherapeutic drugs have been approved for clinical use, and have benefited patients with bladder cancer (Patel et al., 2020), melanoma (Tagliaferri et al., 2022), breast cancer (Keenan and Tolaney, 2020) and lung cancer (Lahiri et al., 2023). However, due to the presence of immunosuppressive TAMs in tumors, which protect cancer cells from treatment-induced cell death, some patients respond poorly to immunotherapy and may even develop highly progressive disease after treatment. However, due to the existence of immunosuppressive TAMs in tumors, which protect cancer cells from treatment-induced cell death, some patients have poor response to immunotherapy, and may even develop into highly advanced diseases after treatment. The positive reaction of immunotherapy usually depends on the dynamic interaction of tumor cells and immunomodulators in the TME (DeBerardinis, 2020). Although immunotherapy targeting the TME is a breakthrough in cancer treatment, the overall effective rate still needs to be improved.

In order to enhance the efficacy of tumor immunotherapy, it is essential to change the tumor microenvironment. The combination of ultrasound and microbubbles regulates the tumor immunosuppressive microenvironment by damaging endothelial cells with generated microfluidics, microfluidics and free radicals, causing microvascular rupture and tumor cell apoptosis, impeding tumor angiogenesis and enhancing the effect of immunotherapy (Alphandéry, 2022; Han et al., 2022; Li et al., 2022). In addition, drugs and exogenous genes can be released targetedly through local ultrasonic irradiation, so as to get hold of better therapeutic curative effect (Ho et al., 2018). Therefore, UTMD shows a excellent application in enhancing the immunotherapy effect. Overview of this article, we summarize the benefits of combined application of ultrasound and microbubbles in immunotherapy of the TME in tumor therapy, and describe the future prospects.

## 2 Overview of ultrasound

As a simple, safe and noninvasive method, ultrasound has been widely used in the diagnosis and treatment of diseases. UTMD is a new treatment method which has been developed in the field of tumor treatment in recent years. It has the advantages of precision, efficiency and safety, and has good repeatability. It is enhanced in drug release and exogenous genes through ultrasonic cavitation effect (Myers et al., 2018; Awad et al., 2021; Wang et al., 2022). The cavitation is an important physical phenomenon. When the ultrasonic pressure reaches a certain threshold, the surrounding liquid quickly fills the small cavity of gas and steam, forming micro bubbles (MBS), also known as cavitation nuclei. These MBs are excited under the sound field, and the dynamic process of continuous vibration, expansion, contraction and even collapse releases instantaneous energy and causes extreme physical phenomena (Han et al., 2017; Aryal and Porter, 2022; He et al., 2022), such as luminescence, high temperature, high pressure, discharge and micro-jet (Snipstad et al., 2021).

**FIGURE 1:**
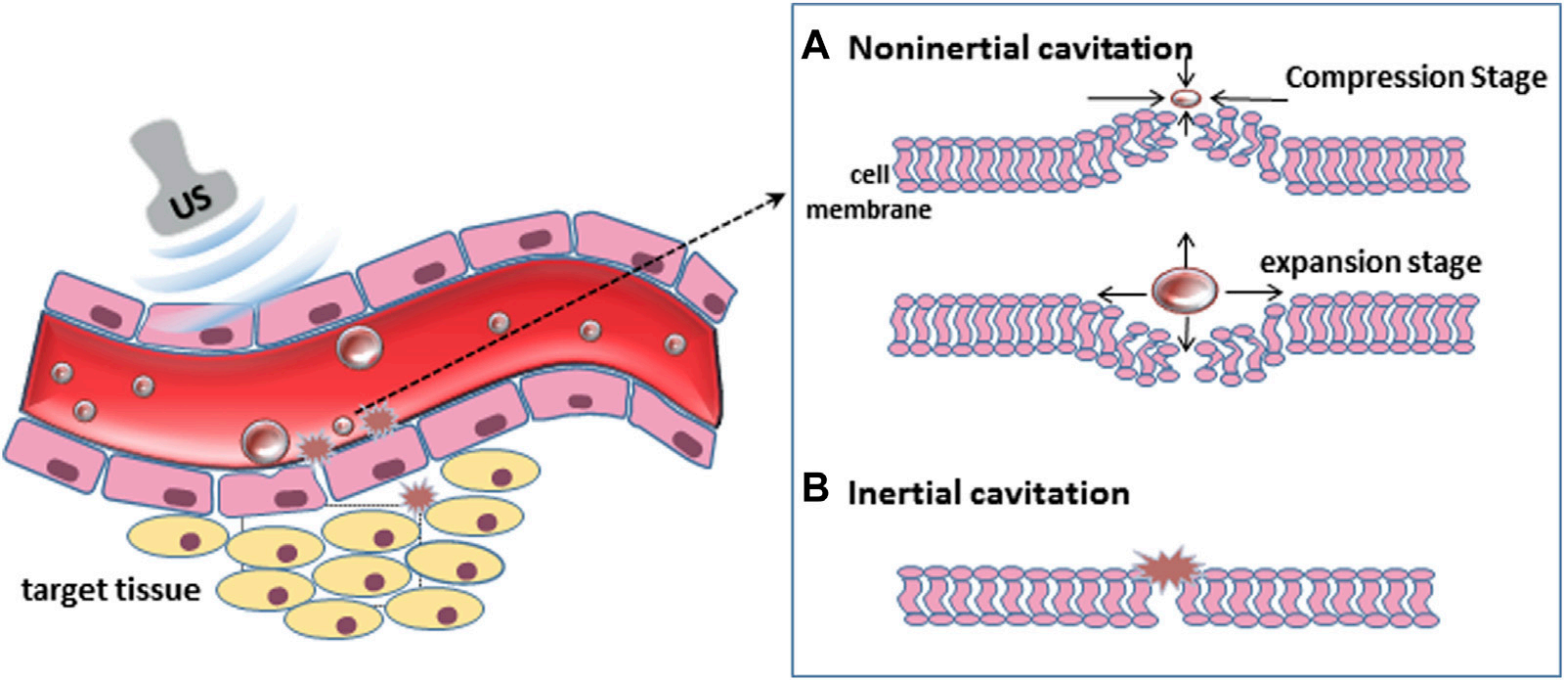
Ultrasonic cavitation effect.

The principle of enhancing ultrasonic cavitation effect is to increase the number of cavitation nuclei by increasing exogenous MBS. With the increase of MBS, The energy required to generate cavitation decreases with the increase in MBS, which results in a decrease in the energy threshold for cavitation effect (Chowdhury et al., 2020) (Figure 1 Ultrasonic cavitation effect). When high-frequency ultrasound is generated, microbubbles will undergo asymmetric oscillation, causing asymmetric expansion and collapse of their volume. The strong compression of gas inside bubbles and the repeated impact of local pressure generated by surrounding fluids have a significant impact on cells or tissues, and will produce thermal effects, which will cause denaturation of various immunoregulatory proteins in tissues (Chen et al., 2021; Entzian and Aigner, 2021) and acting on factors such as VEGF can lead to microvascular rupture in tissues (Mitragotri, 2005; Thomas et al., 2019). In animal models, HIFI was combined with liposomes containing temperature sensitive drugs, and it was found that the permeability of drugs to tumors was significantly enhanced (Sadeghi-Goughari and Kwon, 2019). Due to the low frequency and low intensity of low-frequency ultrasound, under the action of ultrasound, bubbles oscillate uniformly without rupture, resulting in microfluidics characterized by fluid flow (Niu et al., 2020). Microfluids exert shear stress on cells while generating heat, and lead to sound holes which help to open the tissue barrier formed by endothelial cells. Studies have shown that under the action of low-frequency ultrasound, the collapse process of MB caused by cavitation effect produces jets and releases energy, which can instantly rupture adjacent cell membrane, increase their permeability and promote the ingestion of drugs by cells (Dong et al., 2017; Yang et al., 2018). At present, the way of using UTMD to improve the efficiency of drug targeting and delivery in local tissues is widely recognized in research (Feril and Tachibana, 2012; Cai et al., 2022; Yang et al., 2022). Therefore, By combining ultrasound with microbubble therapy, tumor cells can be killed specifically, anti-tumor immune response can be stimulated, and tumor microenvironment can be improved, which is crucial for tumor immunotherapy (Zhang et al., 2017).

## 3 Tumor microenvironment and immunotherapy

**FIGURE 2:**
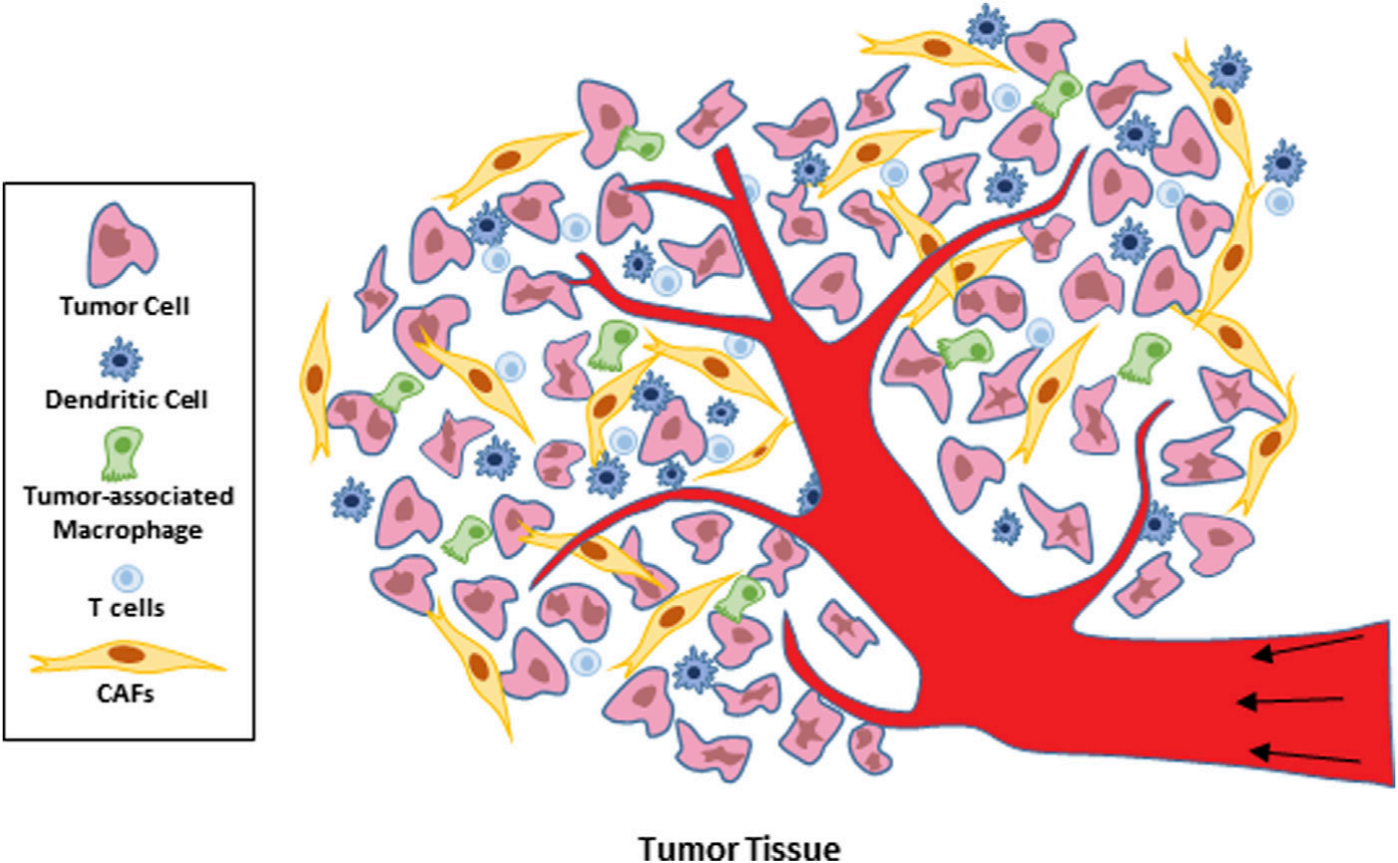
Cell composition with in the tumor microenvironment.

In recent years, researchers have gone deep into the components of the tumor microenvironment and noticed that it is composed of cytotoxic T lymphocytes (CTLs) with natural anti-tumor immunity and cells that inhibit the function of tumor immunotherapy: myeloid derived suppressor cells (MDSCs), tumor associated macrophages (TAMs), and tumor associated fibroblasts (CAFs) (Pitt et al., 2016) (Figure 2 Cell composition within the tumor microenvironment). Therefore, in-depth understanding of the immunosuppressive mechanism of immunosuppressive cells in the TME is of great significance for expanding the idea of immunotherapy and improving the curative effect. Due to the formation of tumor microenvironment, the interstitial pressure of tumor increases, the ability of immune cells to infiltrate tumor tissue is insufficient, and the lack of CTL and other immune cells with antigen stimulation in tumor tissue can cause insufficient effective inhibition of tumor growth and promote tumor immune escape; In addition, The inability to effectively activate antigen-specific T cells that inhibit tumor growth and the poor release of tumor antigens to peripheral lymph nodes leads to the depletion of T cells that are already lacking and insufficient direct or indirect antigen presentation; However, tumor cells and immunosuppressed cells secrete a variety of negative immunoregulatory factors, which lead to immune escape and can not recognize and present tumor antigens. All these factors lead to the formation of an immunosuppressive microenvironment, the clinically observed resistance to immunotherapy, and the tumor promoting effect (Bejarano et al., 2021; Liu et al., 2022). However, at present, in cancer, immunotherapy for effective activation of T cells is mainly a strategy to enhance adaptive immunity by inhibiting immune checkpoints and promoting T cell release (Hope and Salmond, 2019; Ajina et al., 2020; Lim et al., 2020). In recent years, studies have found that t-cell-promoting immune tumors, such as monoclonal antibody trastuzumab (TRA), act on HER2 positive breast cancer and improve antibody cell dependent toxicity. However, as a first-line drug, its drug resistance and targeting are still clinical challenges (Modi et al., 2020; Elamir et al., 2021). Therefore, it is a promising research direction in tumor treatment to seek to improve the anti-tumor efficiency of immune cells such as T cells and reduce the cytotoxicity of drugs.

Dendritic cells are a group of highly heterogeneous professional antigen presenting cells (APCs), which have the functions of ingesting, processing and presenting antigens (Verneau et al., 2020; Zimmermannova et al., 2021). Some studies have shown that cDCs can induce a strong anti-tumor CTL response, and then reduce the tumor growth in mice inoculated with cDC2, which is related to the decrease of MDSCs and reprogramming of TME’s natural tam (Gerhard et al., 2021; Marciscano and Anandasabapathy, 2021). In addition, TME can trigger a variety of mechanisms to interfere with DC function, including reducing and inhibiting the recruitment of dendritic cells in tumors through the production of chemoattractants (such as carbon tetrachloride and CCL5), as well as by reducing the survival signals required for DC differentiation and survival growth factors and IL-12 (Xiang et al., 2022). Together, these two factors lead to insufficient activation of T cells, and may induce T cell to tolerate TAA (Garris and Luke, 2020). The increase in the density of immunocytotoxicity-related dendritic cells in the TME makes the prognosis of ovarian cancer, lung cancer and breast cancer better (Batchu et al., 2021; Ding et al., 2021). Dendritic cells can be regarded as a promising target for cancer immunotherapy.

TAMs is an important part of tumor inflammatory infiltration. In the process of tumor formation, the pool of resident macrophages is expanded by *in-situ* proliferation and supplemented by monocyte-derived macrophages (MDMS) recruited to TME (Mehta et al., 2021; Munir et al., 2021; Koll et al., 2023). However, these TAMs in TME have been further modified, which leads to phenotypic and functional heterogeneity between different tumor types, including bladder cancer, breast cancer and pancreatic cancers (Sun X. et al., 2022; Sun M. et al., 2022; Hezaveh et al., 2022). Several studies have confirmed that TAMs in the TME can regulate cancer progression through various mechanisms, including immune escape, angiogenesis, cancer cell invasion and immunosuppression to promote tumor occurrence and enhance the subsequent inflammatory stimulation of tumor growth and metastasis (Miyake et al., 2016; Bao et al., 2022). In addition, TAMs can affect the poor prognosis, drug resistance and cancer recurrence after routine treatment, for example, due to colony stimulating factor 1 (CSF 1) (Akkari et al., 2020; Garrido-Martin et al., 2020). Therefore, different methods have been developed to target TAMs, including blocking the recruitment and infiltration of TAMs into the TME, inhibiting the induced activation of TAMs on CSF and cytokines such as IL-4 and IL-10, and interfering with the transcription of TAMs function in the TME (Zhang H. et al., 2021; Hezaveh et al., 2022).

In TME, CAFs, as one of the main cell types producing ECM molecules, can support tumor growth through various mechanisms (Ribeiro Franco et al., 2020). CAFs produce matrix remodeling enzymes while depositing into ECM, and play a very important role in tumor invasion, metastasis and drug resistance (Deepak et al., 2020). In addition, CAFs communicates with tumor cells by secreting a variety of cytokines, exosomes and growth factors, and promotes malignant biological behaviors such as proliferation, invasion and metastasis of tumor cells by transmitting signal molecules such as mRNA, and plays an active role in the occurrence and development of tumors (Erdogan and Webb, 2017; Ren et al., 2019; Eckhardt et al., 2020). CAFs also have a greater impact on the vasculature and immune cells in the TME. For example, VEGF secreted and promoted by CAFs can drive angiogenesis (Komi and Redegeld, 2020; Zhao et al., 2022), while secretion of IL 6, CXCL 9 and TGF regulates the T cell responses (Jiang et al., 2017; Yoshida, 2020). At present, in the face of the severe situation of tumor immunotherapy efficacy and drug resistance, CAF can be used as a potential target in tumor diagnosis and treatment, with broad clinical application prospects.

## 4 Mechanism and application of ultrasound combined with microbubbles in tumor microenvironment immunotherapy

### 4.1 Promote antigen release from cancer cells

**FIGURE 3:**
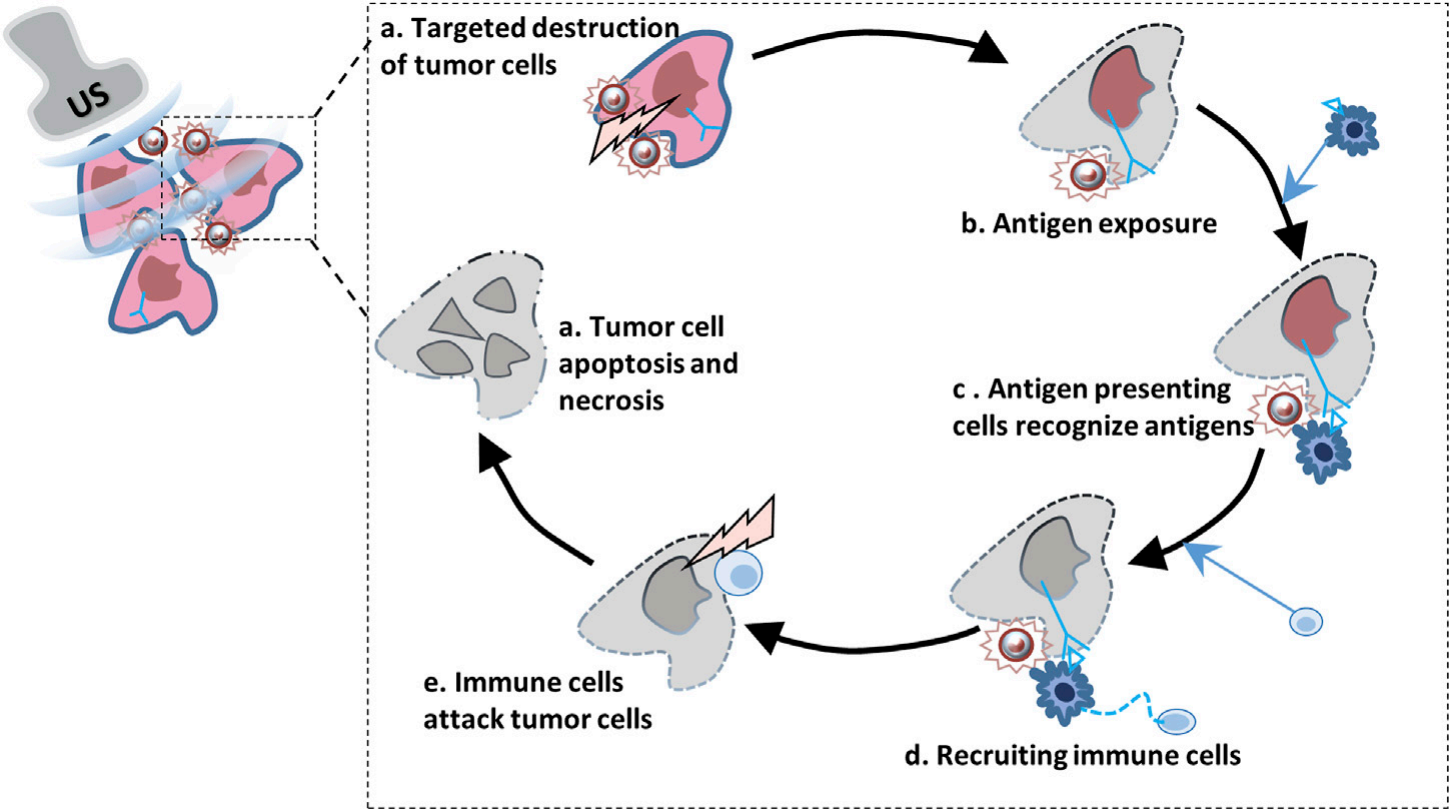
Ultrasound combined with microbubbles promotes the release of cancer cell antigens and damages tumor cells.

The first step of immune clearance of tumor cells is to activate natural killer cells (NK), macrophages and dendritic cells, which can start the inflammatory and immune reactions by recognizing tumor-associated antigens, thus clearing abnormal expression cells (Mollica Poeta et al., 2019; Blass and Ott, 2021; Lahiri et al., 2023). However, the lack of antigen stimulation in tumor immune microenvironment can the growth of tumor cells cannot be effectively inhibited and immune escape. Therefore, how to induce strong and specific tumor antigen release is a problem faced by tumor treatment. 1In recent years, ultrasound, as a safe and effective method to inhibit tumor growth, can affect tumor hypoxia reaction by inducing mechanical and thermal effects such as oscillation and cavitation, and induce tumor cell apoptosis, thus increasing antigen exposure and stimulating immune response (Chen et al., 2014; Giovagnoli-Vicuña et al., 2022) (Figure 3 Ultrasound combined with microbubbles promotes the release of cancer cell antigens and damages tumor cells). It has been reported that UTMD can lead to apoptosis of tumor cells by acting on ceramide signaling pathway and calcium ion level in tissue environment, while tumor cells that are about to die and undergo stress release immune recognition agents, such as damps, to stimulate the body to produce immune response to tumors (Wang L. et al., 2021; Zhu et al., 2021). However, liposomal microbubbles in animal transplanted tumors after ultrasound irradiation, cellular immunity against cancer cells is triggered. Experimental data suggest that tumor associated antigens released from cancer cells damaged by ultrasound irradiation are increased and recognized by DCs. In addition, the anti-tumorffect depends on the intensity of ultrasound exposure, which suggests that the degree of tumor damage may be related to the antitumor immune response (Oda et al., 2012; Xiong et al., 2020; Zhang et al., 2022). In Wang L’s research, liposome microbubbles were used to deliver paclitaxel to glioblastoma xenografts in nude mice and were treated with low frequency ultrasound irradiation. The results showed that low frequency ultrasound combined with microbubbles could induce apoptosis and inhibit cell proliferation (Wang L. W. X. et al., 2021). In the study of Elamir A encapsulating anti-HER2 monoclonal antibody on liposomes to target the treatment of HER2 positive breast cancer, it was reported that low-frequency ultrasound combined with microbubble therapy was more effective than other groups in inhibiting tumor growth, promoting apoptosis, inhibiting cell proliferation and angiogenesis, and enhancing immune response (Elamir et al., 2021). In recent years, it has been found that high-intensity focused ultrasound is used to focus ultrasound energy from the body to the target area, and has been used as an ablation therapy for cancer. This therapy mainly uses the mechanical effect of boiling cathepsin, that is, high-intensity focused ultrasound, to induce cancer cell damage, thus inducing a strong immune response (Aryal and Porter, 2022; Wu Y. et al., 2023). This approach significantly increases the levels of tumor associated antigens, chemokines (IL-8) and a large number of cell damage related molecules, which in turn further stimulated the uptake and presentation of antigens by DCs (Yang H. et al., 2019; Zhang et al., 2022). Therefore, inducing the death of immunogenic cell by external ultrasonic energy may be a potential immunotherapy for cancer.

### 4.2 Promote the presentation of antigen-presenting cells (APCs)

Tumor immunotherapy refers to the proliferation of T lymphocytes *in vivo* stimulated by antigens presented by antigen presenting cells, resulting in specific cytotoxicity, thereby killing pathogens. Tumor cells and immunosuppressed cells secrete a variety of negative immunoregulatory factors in tumor tissues, which leads to the inability of immune cells to recognize and present tumor antigens. The general interruption of antigen presentation interrupts tumor antigen recognition, and the defect of antigen presentation provides an immune escape mechanism for tumor cells in the TME (Bishop, 2016; Jhunjhunwala et al., 2021). The presentation of tumor specific antigens by MHC molecules on APCs (such as DCs) is the key to stimulate specific T cell responses (Yang et al., 2023; Zhu and He, 2023). After MHC antigen is presented to APC, the most critical step is to induce CD 8 + T cell responses specific for cancer cells. In the case of TME, CD 8 + T cells can not be effectively triggered (Alrubayyi and Rowland-Jones, 2023; Yang et al., 2023). Therefore, it is necessary to induce dendritic cells to cross present extracellular antigens in DC-based cancer immunotherapy. According to reports, The combination of ultrasound and microbubbles is a new method of tumor specific antigen delivery. By opening the transient pores in the cell membrane of these cells, tumor specific antigens can be directly delivered to dendritic cells, and then the extracellular antigens can be directly recognized in the cytosol. 3The results showed that the antigen delivery system combined with ultrasound and microbubbles could be used for antigen delivery of tumor immunotherapy based on DC (Pelka and Guha, 2023). In the xenotransplantation mouse models of RM 1 (prostate cancer), MC38 (colon cancer) and B16 (melanoma), ultrasound-mediated cavitation induces tumor cell necrosis, which significantly increases the damage related to molecular mode damping release and tumor antigen presentation, thus making the tumor sensitive to ICB treatment (Hu et al., 2022). Sun W’s experiments on 4T1 homologous mouse model proved that ultrasound sensitizer CE 6 and ultrasound irradiation jointly promoted mRNA escape from endosome, thus enhancing antigen presentation, effectively triggering anti-tumor immunity and preventing tumor metastasis (Sun et al., 2023). In addition, a nano-scale cancer vaccine was designed in this study, which was sealed by Ce6 and other materials based on PLGA (lactic co glycolic acid). After subcutaneous injection, the nano-vaccine can be effectively delivered to antigen presenting cells (APCs) of lymph nodes. As is known to all, tumor vaccines is considered as a promising immunotherapy method by inducing specific anti-tumor immune responses. In order to effectively present tumor-associated antigens and improve tumor immunity, a cell-free DCs vaccine has been developed, in which the cell membrane antigen of breast cancer cells and dendritic cells derived from human PBMC cultured *in vitro* are combined into ultrasound contrast agent microbubbles (MB). The research results show that DC-IMBs can accumulate in lymphoid organs and induce anti-tumormmune responses, and significantly inhibit tumor growth through apoptosis, prolong the survival time of treated animals and exert anti - tumor effect (Jugniot et al., 2022).

### 4.3 Priming of T cells and infiltration of T cells into tumor tissues

The reasons for the weakening of immune clearance mechanism and tumor growth and metastasis in TME are (Crespo et al., 2013; Kishton et al., 2017; Ma et al., 2019):a) the reduced proliferation of immune cells and the insufficient ability of immune cells to infiltrate tumor tissues; b) Antigen specific T cells are heavily depleted and poorly activated. Therefore, in cancer immunotherapy, it is very essential to stimulate antigen-specific T cell responses to directly attack cancer cells. T cell immunotherapy with chimeric antigen receptor (car T cell) is an adoptive cancer immunotherapy (Boulch et al., 2021), which expresses engineered cells of chimeric antigen receptor (CAR) against specific tumor antigens (TA), that is, it is designed according to the principle of T cell receptor and costimulatory signal transduction, and this therapy can identify and eliminate cancer cells. But at present, its main therapeutic target are hematological tumors, such as leukemia and lymphoma (Agarwal et al., 2020; Yang et al., 2021). The reason is that the amount of cardiac T cells infiltrating into solid tumor tissues is very low. Because it is difficult to achieve a sufficiently effective T cell infiltration in solid tumors, Based on the combination of ultrasound and microbubbles, the permeability and function of endothelial cells can be directly affected by the cavitation produced. Under the mechanical damage of us irradiation, the infiltration of cd8 + t cells in tumor tissues increased significantly, which effectively improved the immunosuppressive TME. It also induces systemic anti-tumor immune responses and enhances the therapeutic effect of anti-PD-L 1 antibody in local and distant tumors. Recently, it was reported that UTMD plays an indispensable role in enhancing the transfection efficiency of tumor suppressor genes and cytokines and promoting the recruitment of T cells at tumor targeted sites. We detected its synergistic efficacy in the treatment of tumors with anti-PD-L1 antibody in a mouse tumor model (Ilovitsh et al., 2020; Tang et al., 2023). By combining ultrasound with tumor targeting microbubbles, Hu J team showed that USNB promoted the infiltration and antitumor activity of CD8^+^ T cells (Sun et al., 2023). In addition, studies have shown that the new combination therapy of low intensity focused ultrasound targeted microbubble destruction (LIFU-TMD) and PD-L 1 blockade also shows the same effect of reducing tumor and enhancing T cell infiltration (Wu N. et al., 2023). Furthermore, in order to improve the cure rate of solid tumor based on immune adjuvant, some tumor-associated antigens (TAAs) produced by experimental UMC therapy show vaccine activity, especially in the presence of immune adjuvant, which jointly promoted the maturation of dendritic cells and induced the production of cytokines (Zhang N. et al., 2021). Importantly, UMC can downregulate immune checkpoint molecules, such as CD274, Foxp3 and CTLA4, and cooperate with ally to stimulate the activation and proliferation of T cells *in vivo*, so as to promote tumor treatment (Omata et al., 2001; Suzuki et al., 2010; Hunt et al., 2015). In enhancing HIFU therapy, antigen-sensitized DC prepared by ultrasound and microbubbles can cause a large number of proliferation of T cells and CTL, which has strong anti-tumor effects (Singh et al., 2019; Ye et al., 2022). In summary, the combination of ultrasound and microbubbles can open the tight connection between endothelial cells, induce the secretion of chemokines, promote the infiltration, proliferation, and activation of cytotoxic T cells, and infiltrated immune cells such as T cells and lymphocytes can improve the microenvironment of tumor tissue for further activation of anti-tumor immune responses.

### 4.4 Enhancing the recognition and killing effect of T cells on cancer cells

When the microenvironment of tumor tissue is in immunosuppression state, it can inhibit the activation of T cells mediated by T cell receptor (TCR), thus inhibiting the activation of effector T lymphocytes, increasing the expression of CD8+T cells through PD-1/PD-L1 signaling pathway, and finally leading to the immune escape of tumor (Jiang X. et al., 2019; Jiang Y. et al., 2019; Yi et al., 2022). In order to improve this environment, it has obtained clinical approval or is being evaluated in trials, including immunotherapy, anti-antigenic drugs, and immune checkpoint blockade (ICB). However, due to the existence of the microenvironment of immunosuppressed tumors, the clinical therapeutic effect of some patients is limited (Bader et al., 2020). As a new tumor treatment method, ultrasound combined with microbubbles has become a hot spot in tumor immunotherapy research because of its advantages of targeting, safety and high efficiency. In a mouse colon cancer model treated with ultrasound combined with microbubble (Tang et al., 2021), tumor perfusion, CD8+T cell infiltration and anti-PD-L1 antibody delivery were also observed to increase. In particular, flow cytometry showed that ultrasound combined with microbubble treatment not only increased the infiltration of CD8+T cells into tumors, but also promoted the expression of apoptosis indexes such as Ki67, which had a stronger killing effect on tumor cells and more obvious inhibitory effect on tumor growth. In addition, NK cells are essential for T cells to mediate tumor immunity. Studies have found that the clearance of NK cells also caused a reduction in the recruitment and activation of tumor specific CD8+T cells (André et al., 2018; Yu et al., 2021). Sta Maria et al. reported (Sta Maria et al., 2015), in the xenograft tumor of human colorectal adenocarcinoma mice, compared with the group that did not receive ldbFUS treatment, the group that injected MBs + NK cells into the tail vein of mice and underwent ultrasound irradiation had significantly more NK cell aggregation. The efficiency of adoptively transferred natural killer cells (NK) in treating TME of solid tumors is hindered. In the experiment of enhancing the adoptive penetration of NK-92MI cells into ovarian tumors by using the combination of focused ultrasound (FUS) and microbubbles, NK cell are targeted to activate and release IL-2 to maintain their viability, so as to increase NK cell-mediated tumor cell killing (Yang C. et al., 2019). In conclusion, the combined treatment of ultrasound and microbubbles promoted the recognition of tumor cells by T cell receptors and led to their death, and enhanced the killing effect of T cells in the tumor immunosuppressive microenvironment. In the near future, we may apply ultrasound and microbubble technology to cancer immunotherapy to achieve ideal results.

### 4.5 Targeted therapy for tumor vasculature

In the TME, the high proliferation rate of ECs, the decrease of cell tight junctions, the abnormality of peripheral cell coverage and the increase of ECM deposition lead to the abnormality and dysfunction of the tumor vascular system, which provides the conditions for the growth, proliferation, invasion and metastasis of tumors. At present, the use of effective vascular therapy for anti-tumor has been extensively studied. In the past clinical studies, the application of VEGF pathway inhibitors showed more effective anti-tumor effects, such as several inhibitors targeting VEGF pathway (Ranieri et al., 2006; Costache et al., 2015; Kim et al., 2022), including bevacizumab and gemcitabine. In the experiment of pancreatic cancer in mice, blocking BICC1/LCN2 signal pathway can reduce the microvessel density and tumor volume of mouse PAAD cell grafts, enhance the antitumor effect of gemcitabine, and delay tumor growth (Kooiman et al., 2020). In mouse models of breast cancer and neurocytoma, thalidomide, an anti-vascular drug, is related to the reduction of angiogenesis and the inhibition of tumor growth and metastasis (Wan et al., 2021; Al Kawas et al., 2022; Huang et al., 2023). However, blocking VEGF pathway by antibodies to inhibitors such as VEGF and VEGFR, as a tumor inhibition therapy, has poor curative effect, and even tumor resistance is a common event in patients. Therefore, safe and effective improvement of tumor vessel normalization and enhancement of the concentration and efficacy of VEGF targeted therapeutics are expected to become an important part of anti - antigenic therapy (Heath and Bicknell, 2009). Ultrasound combined with microbubbles to enhance the treatment of anti-VEGF and anti-VEGF receptor antibody is helpful for immunosuppression in the tumor microenvironment (Curley et al., 2017). Based on previous studies, ultrasound combined with microbubbles has the ability to improve the composition and integrity of tumor vascular system, improve drug permeability and normalize blood vessels, which is one of the main clinical strategies to improve the drug delivery and treatment effects of tumor TME (Kooiman et al., 2020). For example, in human breast cancer cells, ultrasound mediated disruption of VEGF targeting and paclitaxel (PTX) - loaded lipid microbubbles (VTPLLM + US), VTPLLM + US inhibited MCF-7 cell proliferation and accelerated apoptosis by regulating VEGF expression (Su et al., 2019). In a study of gene therapy for breast cancer, the same results showed that VEGF-CNA could effectively inhibit the expression of VEGF-C protein and mRNA in MCM cf-7l and significantly inhibit the growth of human lymphatic endothelial cells (LECs) and MCF-7 cells *in vitro* (Xu et al., 2013). In addition, in the study of prostate cancer (PCa), low-frequency ultrasound combined with microbubbles stimulated DCs in the bone marrow of BALB/c mice by adding cytokines (granulocyte macrophage colony stimulating factor (GM-CSF) and interleukin −4 (IL-4)). Cell migration and invasion tests and cell enhancement tests showed that downregulation of VEGF expression inhibited the evolution of PCA cells, and it can be inhibited by VEGF (Zhang et al., 2017). Therefore, ultrasound mediated microbubble destruction (UMMD) plays a key role in the targeted treatment of tumor blood vessels, inhibiting tumor proliferation and enhancing the efficacy of cancer immunotherapy, and may be a powerful and promising technology.

## 5 Regulation of tumor immune microenvironment by UTMD

Clinical studies have found that the tumor immunosuppression microenvironment plays a key role in the development and treatment response of tumor disease, and is also the main reason for drug resistance and off-target toxicity of immunotherapy. 2Recent studies have found that ultrasound-targeted microbubble sonodynamic therapy (SDT) is a promising strategy to inhibit tumor growth and activate immunotherapy against tumor immune response by inducing microvascular rupture and tumor cell apoptosis to regulate tumor immunosuppression microenvironment, block tumor angiogenesis and enhance the effect of immunotherapy (Kim et al., 2020; Abe et al., 2022; Han et al., 2022). Team Ji C developed ultrasonic microbubble nanoparticles with mitochondrial targeting and sound sensitizer (IR780), which were wrapped in perfluoronalKane (FDC). After mitochondrial targeting and mitochondrial dysfunction in 4T1 tumor-bearing mice, the tumor growth *in vivo* and *in vitro* was inhibited, and the immunogenic cell death was enlarged, which led to the effective improvement of the microenvironment of immunosuppressed tumors, the maturation of dendritic cells and the increase of the number of infiltrating immune cells, which further enhanced the immune response to tumor immunotherapy (Ji et al., 2021). In addition, the ultrasonic cavitation effect exhausted tumor blood perfusion through abnormal blood vessel rupture, induced TME to transform and sensitize anti-PD-l immunotherapy, induced some cellular immunogenic cell death (ICD), and significantly inhibits the growth of mouse 4T1 breast cancer. Moreover, it was also found that the levels of dendritic cells (DCs) and CD8^+^ T cells in tumor tissues increased significantly (Wu N. et al., 2023). More than that, UTMD and small molecule ribonucleic acid have the potential to interfere with the immune system. In the study of hepatocellular carcinoma (HCC) mouse model, UTMD-microRNA combined treatment delivered microRNA-122 and anti-microrna-21 into HCC tumors, which reduced the level of GM-CSF and affected the expression of cytokines in the immune microenvironment, thereby improving the therapeutic effect on liver cancer (Wischhusen et al., 2020). To sum up, the combined application of ultrasound and microbubbles can transform the tumor microenvironment into a state conducive to the infiltration of immune cell, thus promoting the anti-tumor immune response. Therefore, this strategy has the potential to be a tumor immunotherapy based on tumor microenvironment immunomodulation.

## 6 Expectation

The tumor immunosuppressive microenvironment is the main cause of immune therapy resistance and off target toxicity observed in clinical practice. Although various anticancer drugs and methods have been developed for immunosuppression environment, most cancer treatments involve tumor cells escaping from immune attacks, and drugs can not completely reach the target tumor cells and aggregate to form a killing effect. Adopting a safe, targeted, and effective approach to the treatment of tumor microenvironment has become an important direction for researching and conquering tumors. Ultrasound combined with microbubble therapy is an emerging tumor treatment strategy that has the characteristics of enhancing vascular permeability, non-invasive, and selectivity. In fact, multiple clinical studies have begun to explore the tumor microenvironment of different target organs. At present, a number of clinical studies on solid tumors, such as breast cancer, prostate cancer and pancreatic cancer, are in progress. Focused ultrasound combined with microbubbles can improve tumor vascular permeability, limit the enhancement of anti-tumor immunity in the tumor microenvironment, and is suitable for the treatment of primary tumors. Several articles reported that in order to improve the stability and efficiency of drug delivery and reduce the interference of TME on drugs, targeted microbubbles were used to deliver drugs to tumor cells, thus improving the efficiency. In addition, mechanical fluctuant and thermal effects generated by UTMD are also the research focus of tumor immunotherapy. This combination can release cytokines and enhance the infiltration of immune active cells into tumor tissues, thus achieving effective cancer treatment. In addition, UTMD can induce cancer cells to release antigens and dangerous signals, enhance the induced anti-cancer immune response, and begin boosting cell membrane permeability and causing damage to tumor cells together to initiate effective anti-tumor immunity. Therefore, further study on the effect of ultrasound combined with microbubbles on the tumor microenvironment can provide new ideas for tumor immunotherapy and new development direction for tumor targeted therapy with high efficiency and low side effect.

## 7 Conclusion

The combined action of ultrasound and microbubbles can induce a variety of biological responses through mechanical fluctuant and thermal effects. TME plays an important role in dynamically regulating the progress of cancer and influencing the therapeutic results. The strategy of taking the TME as a therapeutic target is a promising cancer treatment method that has emerged in recent years. Therefore, the importance of UTMD in the regulation of tumor immune microenvironment has been widely recognized now. Ultrasound and microbubbles work together to induce antigen release of tumor cell through mechanical or thermal effects, promoting antigen presentation and T cells’ recognition and killing effect on tumor cells, and improve tumor immunosuppression microenvironment, which will be a breakthrough to improve the drug resistance, insufficient targeting and toxic and side effects of traditional treatments such as ICB and CAR-T cell therapy. In order to improve the efficacy and immune regulation of TME targeted tumor therapy, it is essential to develop and optimize microbubble ultrasound application systems for organs or diseases. However, improving the targeted curative effect and immune regulation effectiveness of tumor treatment is also one aspect that needs to be improved in ultrasound combined with microbubble therapy. In addition, there is no unified international standard for ultrasound parameters, microbubble types, doses, etc. That need to be applied in tumor experiments, and it needs to be further improved. Therefore, UTMD in the field of TME will continue to focus on developing more effective strategies to regulate the immunosuppression mechanisms, so as to activate anti-tumor immunity and/or improve the efficacy of immune-targeted drugs, which have great potential in targeted treatment of TME and to provide a cutting-edge selection idea and synergistic treatment method for clinical treatment of tumor.
